# Footprints in the Sand: Deep Taxonomic Comparisons in Vertebrate Genomics to Unveil the Genetic Programs of Human Longevity

**DOI:** 10.3389/fgene.2021.678073

**Published:** 2021-06-07

**Authors:** Stephen Treaster, David Karasik, Matthew P. Harris

**Affiliations:** ^1^Department of Orthopaedics, Boston Children’s Hospital, Boston, MA, United States; ^2^Department of Genetics, Harvard Medical School, Boston, MA, United States; ^3^Azrieli Faculty of Medicine, Bar-Ilan University, Ramat Gan, Israel; ^4^Marcus Institute for Aging Research, Hebrew SeniorLife, Boston, MA, United States

**Keywords:** phylogenomics, vertebrates, lifespan, longevity, evolution, GWAS

## Abstract

With the modern quality, quantity, and availability of genomic sequencing across species, as well as across the expanse of human populations, we can screen for shared signatures underlying longevity and lifespan. Knowledge of these mechanisms would be medically invaluable in combating aging and age-related diseases. The diversity of longevities across vertebrates is an opportunity to look for patterns of genetic variation that may signal how this life history property is regulated, and ultimately how it can be modulated. Variation in human longevity provides a unique window to look for cases of extreme lifespan within a population, as well as associations across populations for factors that influence capacity to live longer. Current large cohort studies support the use of population level analyses to identify key factors associating with human lifespan. These studies are powerful in concept, but have demonstrated limited ability to resolve signals from background variation. In parallel, the expanding catalog of sequencing and annotation from diverse species, some of which have evolved longevities well past a human lifespan, provides independent cases to look at the genomic signatures of longevity. Recent comparative genomic work has shown promise in finding shared mechanisms associating with longevity among distantly related vertebrate groups. Given the genetic constraints between vertebrates, we posit that a combination of approaches, of parallel meta-analysis of human longevity along with refined analysis of other vertebrate clades having exceptional longevity, will aid in resolving key regulators of enhanced lifespan that have proven to be elusive when analyzed in isolation.

*Thou know’st ’tis common; all that lives must die*,

*Passing through nature to eternity.*

Shakespeare, W. *Hamlet*.

## Longevity and Aging – Separate Metrics of Extent and Quality

The drive to understand why we have a limited license in life has permeated scientific and artistic thought for millennia. Although lifespan has obvious heritable components, the effect of environmental factors and extrinsic mortality factors shape a complex scenario for which clear answers of the regulation of longevity have been difficult to distill. With the discovery of genetic factors underlying aging in experimental laboratory models, forays into the genetic regulation of these properties have rapidly expanded, uncovering conserved mechanisms across diverse metazoa that influence expression of aging phenotypes and lifespan. Yet, the story gets muddled in that these factors are often quite pleiotropic, having broad roles in normal development and physiology of organisms. To date there has not been a singular defining mechanism or factor specifying how and why we age.

**FIGURE 1:**
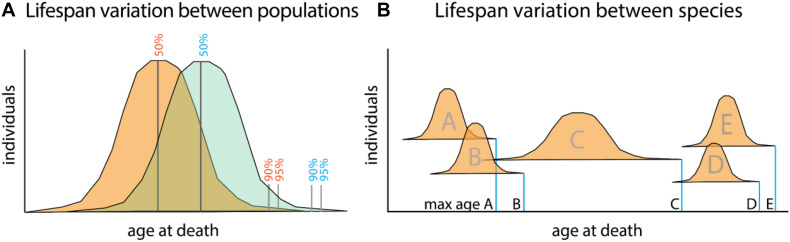
Relationship between aging and lifespan variation versus species defining lifespan. **(A)** Lifespan comparisons within species, measured as mean (50%) or portion of a population living till extended limits of lifespan (90–95%). Differences between populations (orange and green) can identify specific genetic or environmental changes associating with long life. These factors promote viability and often associate with increasing healthspan. Mutant analysis within a particular model organism often encompasses these types of changes as it relates to lifespan. **(B)** Maximum lifespans recorded for different species (A–E). While lifespan variation within a species is capped to a certain extent, variation between species can range dramatically. Changes to maximum lifespan often are associated with protective mechanisms for genomic and genetic fidelity as well as life history changes as they relate to maturation and reproduction.

The difficulty in parsing the generalized regulation of longevity is in part rooted in the difficulty dissociating aging and longevity as distinct processes. Aging, or the progressive deterioration of homeostasis with time, is a component of longevity as increased aging phenotypes often lead to shortened lifespan (Figure 1A). Conversely, the abrogation of many age-related diseases promotes longer life. Longevity, or the propensity for a particular lifespan, is in itself a separate property. This can be best seen in the variation of average lifespans of different species (Figure 1B). Variation in lifespans across species easily dwarfs changes observed within populations due to changes in environment and behavior.

To date, the mechanisms underlying longevity remain unclear. The present-day lifespan of humans is higher than it has ever been before. The increase in global lifespan does not, necessarily, correspond to increased healthspan. Increased longevity still means an increase in the burden of late-life diseases, since advanced age is the greatest risk factor for most chronic debilitating diseases. Hence understanding the integration of factors affecting lifespan and healthspan as they relate to longevity has critical implications.

Here, we review advances in genomic analysis within and across species to help refine the genetic foundations of age-associated diseases and longevity. As such, independent evolutionary occurrences of this species-specific lifespan change can empower comparative approaches to refine the shared mechanisms associating with longevity phenotypes. These evolutionary-refined gene sets can then be leveraged to focus statistical analysis within human cases of extreme longevity to discover core mechanisms of regulation.

## Analysis of Human Variation in the Genetic Control of Longevity

Heritability studies have convincingly demonstrated that at least some fraction of human lifespan is heritable. In tandem, large-scale genome-wide association studies (GWAS) have identified numerous loci associated with age-related traits (Buniello et al., 2019). While genetic studies have functionally shown an inverse effect of multiple age-related, disease-associated variants on lifespan regulation, the number of well-replicated longevity-conferring variants remains limited to variants in *APOE* (*ApoE* ε2), and more recently, *CDKN2A/B* and *IL6* (see Table 1). To date, studies in humans have been hampered by the specific phenotype definitions used, sample sizes of the extreme phenotypes, and modest heritability of the longevity-related traits (Breitbach et al., 2019). This is due to the complex interplay of biological and social factors involved in human aging, as well as the limited power of GWAS, which require sampling thousands of subjects to achieve statistical significance (Breitbach et al., 2019). Genetic studies of aging have also been hindered by an inconsistent use of definitions of aging (reviewed in Baghdadi et al., 2020). The two main ways of conducting research on the genetics of longevity in human populations are by studying (i) the lifespan (continuous trait, years lived) and (ii) the longevity (dichotomous trait, i.e., being among the longest-lived individuals within a specific population). These complexities have limited the resolution and capability of broad association studies of human longevity. Importantly, these genomic analyses focus on a shift of survival in a population; these variables may be genetically distinct from the mechanisms establishing potential for longevity overall (Figure 1A). We argue that an understanding of this shift in lifespan as well as genetic mechanisms of regulating a species specific ‘set points’ (Figure 1B) will aid in the conceptual distinction of aging and longevity in humans.

**TABLE 1:** Genes/loci identified by genome-wide association studies of longevity and lifespan traits.

Mapped gene(s)	Chr.locus	Trait mapped	Associated phenotypes/GWASs	References
FPGT-TNNI3K	1p31.1	Parental longevity A		Pilling et al., 2017
CELSR2 – PSRC1	1p13.3	Parental longevity B, C, D	Cardiometabolic	Pilling et al., 2017
MAGI3	1p13.2	Parental lifespan	Autoimmune	Timmers et al., 2019; Wright et al., 2019
RC3H1	1q25.1	Longevity		Wright et al., 2019
RABGAP1L	1q25.1	Longevity		Wright et al., 2019
KCNK3	2p23.3	Parental lifespan	Cardiometabolic	Timmers et al., 2019
AC012593.1 – SMIM7P1	2p22.3	Parental lifespan		Joshi et al., 2017
IP6K1	3p21.31	Parental longevity H	Heel BMD; hand grip strength	Wright et al., 2019
SLC4A7	3p24	Longevity	Cardiovascular disease	Timmers et al., 2019
HTT	4p16.3	Parental lifespan	Neurodegenerative	Timmers et al., 2019
LINC02513	4p14	Longevity	Heel BMD; blood pressure	Deelen et al., 2019
LINC02227	5q33.3	Longevity (90 years and older)	Heel BMD	Pilling et al., 2017
POU5F1	6p21.33	Parental longevity C, F		Pilling et al., 2017; Wright et al., 2019
AL645933.5	6p21.33	Parental longevity C, D	Musculoskeletal; autoimmune	Joshi et al., 2017
HLA-DRB1 – HLA-DQA1	6p21.32	Parental lifespan	Autoimmune	
		Parental longevity C, F		Broer et al., 2015
AL357139.2 – AL357139.1	6q16.3	Longevity (90 years and older)		Pilling et al., 2017
BEND3	6q21	Parental longevity A, G		Broer et al., 2015
FOXO3	6q21	Longevity	Macular degeneration	Wright et al., 2019
IGF2R	6q25.3	Parental longevity D		Pilling et al., 2017
SLC22A2 – SLC22A3	6q25.3	Parental longevity C	Cardiometabolic	
		Parental longevity A		Pilling et al., 2017
LPAL2	6q25.3	Parental longevity C		Joshi et al., 2017; Pilling et al., 2017
LPA	6q25.3	Parental lifespan		Wright et al., 2019;
		Parental longevity A, B, F, G		Pilling et al., 2017
AL109933.2 – AL109933.1	6q26	Parental longevity C, D, H		Pilling et al., 2017
AL078602.1	6q26	Parental longevity E		Pilling et al., 2017
AP5Z1	7p22.1	Parental extreme longevity (95+)		Pilling et al., 2017
IL6	7p15.3	Longevity	Asthma (age of onset); Cardiometabolic; Multiple sclerosis	Deelen et al., 2019;
		Longevity (>99%)		
		Longevity (> 90%)		Wright et al., 2019
POR	7q11.23	Parental longevity I		Yashin et al., 2010
CYP51A1, AC000120.3, AC000120.4	7q21.2	Lifespan		Wright et al., 2019
LPL	8p21.3	Parental longevity D	Cardiometabolic	Pilling et al., 2017; Wright et al., 2019
GULOP	8p21.1	Parental longevity C, D	Cardiometabolic, smoking, AD	Wright et al., 2019
AC090281.1 – AC008066.1	8p12	Longevity		Pilling et al., 2017
TOX	8q12.1	Parental longevity B, H		Pilling et al., 2017
CDKN2B-AS1	9p21.3	Parental lifespan	Cardiometabolic, cancer	Timmers et al., 2019; Wright et al., 2019;
		Parental longevity C, E, G, H, I		Pilling et al., 2017
AL353615.1 – SOCS5P2	9q34.3	Parental extreme longevity (95+)		Yashin et al., 2010
ECHS1	10q26.3	Lifespan		Wright et al., 2019
AC068205.2, AC068205.1; HSD17B12	11p11.2	Longevity		Wright et al., 2019
FADS1	11q12.2	Exceptional longevity	Age-related diseases, mortality and associated endophenotypes; skin aging	Timmers et al., 2019
FGD6	12q22	Longevity	Cardiovascular; macular degeneration	Pilling et al., 2017
ZW10	11q23.2	Longevity Parental longevity B	Heel BMD	Yashin et al., 2010
MFRP	11q23.2	Lifespan		Pilling et al., 2017
USP2-AS1	11q23.2	Parental longevity G		Pilling et al., 2017
LINC01405	12q24.11	Parental longevity C		Pilling et al., 2017
CUX2	12q24.11	Parental longevity C		Pilling et al., 2017; Wright et al., 2019
ATXN2, SH2B3	12q24.12	Parental longevity C, D, F, G, I	Cardiometabolic, cancer, autoimmune	Timmers et al., 2019
ATXN2-AS – BRAP	12q24.12	Parental lifespan	Drinking behavior, cancer	Pilling et al., 2017
		Parental longevity C	Autoimmune	
ALDH2 – MAPKAPK5-AS1	12q24.12	Parental longevity C		Pilling et al., 2017
NAA25	12q24.13	Parental longevity C		Pilling et al., 2017
TRAFD1 – HECTD4	12q24.13	Parental longevity C		Pilling et al., 2017
PTPN11	12q24.13	Parental longevity C		Yashin et al., 2010
RIMBP2	12q24.33	Lifespan		Yashin et al., 2010
ANKRD20A9P	13q11	Longevity		Pilling et al., 2017
LINC00355 – LGMNP1	13q21.31	Parental longevity F		Pilling et al., 2017
PROX2, YLPM1	14q24.3	Parental longevity C		Yashin et al., 2010
NRDE2	14q32.11	Lifespan		Wright et al., 2019
SEMA6D	15q21.1	Parental longevity D	Smoking-related	Pilling et al., 2017
AC023905.1, SEMA6D	15q21.1	Parental longevity B	Heel BMD; hand grip strength	Pilling et al., 2017
IREB2	15q25.1	Parental longevity C		Timmers et al., 2019; Wright et al., 2019
HYKK	15q25.1	Parental lifespan	Smoking-related	
		Parental longevity I		Pilling et al., 2017
CHRNA5	15q25.1	Parental longevity G I		Wright et al., 2019
CHRNB4	15q25.1	Parental longevity D H		Timmers et al., 2019
FURIN	15q26.1	Parental lifespan	Cardiometabolic; smoking	Pilling et al., 2017;
		Parental longevity C		Timmers et al., 2019
DHODH – TXNL4B	16q22.2	Parental lifespan	Cardiometabolic; dysostoses	Yashin et al., 2010
TLK2, AC008026.1	17q23.2	Lifespan		Pilling et al., 2017
MC2R	18p11.21	Parental longevity G		Timmers et al., 2019
SMARCA4 – LDLR	19p13.2	Parental lifespan	Cardiometabolic	Timmers et al., 2019
LDLR	19p13.2	Parental longevity D		Deelen et al., 2019
TOMM40	19q13.32	Longevity		Pilling et al., 2017; Deelen et al., 2019
APOE, APOC1	19q13.32	Multiple	Cardiometabolic, dementia	Pilling et al., 2017
EXOC3L2, MARK4	19q13.32	Parental longevity B		Pilling et al., 2017
AL050403.2	20p12.2	Parental longevity B		Pilling et al., 2017
CHRNA4	20q13.33	Parental longevity B		Pilling et al., 2017
PARVB	20q13.31	Parental longevity E		Pilling et al., 2017

*(A) Combined parental age at death. (B) Father’s attained age. (C) Combined parental attained age, Martingale residuals. (D) Father’s age at death or father’s attained age. (E) Mother’s age at death. (F) Mother’s attained age. (G) Longevity (both parents in top 10%). (H) Mother’s age at death or mother’s attained age. (I) Father’s age at death. BMD, bone mineral density.*

### Heritability of Lifespan in Humans

The two main designs that have been used to study the heritability of aging are twin studies and genealogical studies. The main advantage of the twin study design is the ability to discriminate between the effect of genetic, shared and non-shared early environmental influences on a trait. However, the effect of adult environment – not captured by this design – can have a significant effect on the phenotype as well (McGue et al., 2014). On the other hand, a classical pedigree/genealogical study design has the advantage of having access to a much larger sample size, especially for the older members of a population. With large and multi-generational pedigrees, one can investigate a traits’ genetic inheritance pattern and delineate between additive and non-additive components of heritability (Karasik et al., 2004).

Twin studies have shown that the heritability of lifespan ranges between 0.01 and 0.27 in various European populations (Ljungquist et al., 1998; van den Berg et al., 2017). Large genealogical studies are more powered to address questions such as to what extent non-additive genetic variance contributes to the heritability of lifespan. Thus, in more than 3 million pairs of relatives, Kaplanis et al. (2018) found that the additive component of lifespan’s heritability was 0.16 (comparable to twin studies), while there was only a mild effect of the non-additive component of heritability (∼0.04). Ruby et al. (2018) using an impressive dataset consisting of hundreds of millions of historical individuals showed a similar heritability of lifespan. The study on the heritability of “longevity” performed in twins by Ljungquist et al. (1998) found that the heritability of longevity was higher in women and increased with advancing age.

Some of the most interesting individuals that may shed reveal secrets of longevity originate from multigenerational, longevity-enriched families, since such families have propensity to be long-lived, but also seem to evade age-related morbidity. Several genealogical studies of long-lived families evidenced that parental longevity could be considered a proxy for lifespan. Long-lived parents have a high probability to beget long-lived offspring, which gives an indication that longevity is indeed heritable (van den Berg et al., 2017). Notably, members of long-lived families have an interesting phenotype beyond extended lifespan, as they seem to be escaping or delaying age-related disease and show a compression of late life morbidity (extended healthspan). Unraveling the genetics of these individuals might help identifying novel mechanisms involved in healthy aging that can subsequently be targeted by therapeutic interventions. An important drawback of longevity research is the arbitrary age thresholds that often were used to signify an extreme age (Baghdadi et al., 2020). In the pre-GWAS era, the age-thresholds used to define longevity were relatively low (i.e., reaching an age above 80 or 85 years) and the sample size was limited. van den Berg et al. (2019) used two independent multi-generational genealogical datasets to determine the most optimal definition of longevity. They found that the strongest heritable component of longevity is present in individuals belonging to the top 10% survivors of their birth cohort with equally long-lived family members (reviewed in Baghdadi et al., 2020).

Altogether, the twin and genealogical studies have shown that human lifespan is heritable, but is significantly influenced by non-heritable factors, which may explain why genetic studies of lifespan have proven to be challenging.

## Genetic Studies of Human Aging

There have been many different approaches to study the genetics of human aging, including candidate gene analysis, linkage and GWAS.

### Linkage Analysis

Linkage analysis studies have used a family based design to identify regions in the genome associated with lifespan/longevity; done in relatively small populations, over the previous decades they helped to identify population-specific loci with large effect on longevity. Many times, these approaches are biased toward *a priori* knowledge of a quantitative trait locus (QTL) associated with aging processes.

The limited genetic diversity in the available pedigrees is likely an explanation for the variability of linked traits identified between different studies. Beekman et al. (2013) found four regions that exhibit linkage with longevity, of which only one could be explained centering near ApoE ε2 and ApoE ε4 alleles, commonly associating with aging and lifespan. This study included 2118 sibling pairs, which is not the most optimal design for linkage analysis. Indeed, the power of the genome-linkage studies to localize QTLs increases with extended pedigrees over nuclear families and sibling pairs/trios, even with the same overall sample size (Blangero et al., 2003).

### Candidate Gene Studies

In an approach driven by knowledge of biological candidate genes, variant(s) in single or several genes, usually those that were pointed out by animal models and progeric syndromes in humans, are studied for association with the aging or longevity phenotype. Such candidate gene approaches, of which an overview can be found in the LongevityMap database^1^ (Tacutu et al., 2018), confirm only two loci that have withstood replication and validation. The first locus is *APOE*, already identified more than two decades ago (Schächter et al., 1994). The second locus is *FOXO3*, which has been functionally validated (Flachsbart et al., 2017; Grossi et al., 2018); interestingly, until recently (Timmers et al., 2020) *FOXO3* was not detected by GWAS so the strength of this locus on longevity in populations is generally not known. In general, biological candidate genes proposed at earlier stages of genetic exploration as “positive controls” have generally not been confirmed by subsequent large GWAS of the same phenotypes.

### Genome-Wide Association Studies

The GWAS approach is suitable to detect the effect of common genetic variation (usually defined as frequency of minor allele ≥ 1%) on a trait of interest. To date, this approach has resulted in the identification of thousands of loci underlying individual human traits and diseases. However, GWAS of longevity to date have provided only a handful of genome-wide significant loci (GWS, usually considered as *p*-value less than 5^∗^10^–8^). The first lifespan-related GWAS, based on age at death (Walter et al., 2011), which assembled a relatively low sample size, did not identify any GWS loci. The most consistent evidence is obtained for variants in the *APOE* gene (Partridge et al., 2018), Table 1. However, copy number variation (CNV) studies (Kuningas et al., 2011; Nygaard et al., 2016) have shown association with extended lifespan, such as a duplication on 7p11.2 (Zhao et al., 2018).

The recent emergence of the UK Biobank has significantly enhanced research on the genetics of lifespan. The most recent effort using parental lifespan data from this databank, as well as several additional studies in the LifeGen initiative, has resulted in the identification of 12 loci that passed threshold for genome-wide significance (5^∗^10^–8^). Many of the loci have previously been associated with age-related diseases, including cardiometabolic, autoimmune and neuropsychiatric diseases – all underlying major death causes – which likely explains their association with lifespan in this study (Timmers et al., 2019).

### Using Specific Phenotypes to Refine Genetic Association in Human Longevity

Genetic association studies have been less successful for diseases in which phenotypes are difficult to quantify and to standardize among discovery and replication cohorts such as behavioral traits and mental-health-related diseases. Likewise, progress in the aging field is slowed by a vague definition of the phenotype (Sebastiani et al., 2016; Partridge et al., 2018). It has been proposed that research into the genetics of longevity may benefit from the ongoing efforts to study the genetics of healthspan and age-related disease-informed GWAS (Zwaan et al., 1995; McDaid et al., 2017). Studies such as those that systematically dissect musculoskeletal and physical function, including hand grip strength (Willems et al., 2017; Tikkanen et al., 2018) and gait speed (Ben-Avraham et al., 2017) could give insight into healthy aging. Intuitively, more objective definitions, such as restricting cases to the 10% longest lived individuals within a population (van den Berg et al., 2019), should also be promising. Such genetic studies can use country-, sex- and birth cohort-specific life tables within a population the cases were derived, although the sample size of such studies will likely be small due to limited availability of genetic data on such individuals worldwide. As an example, recent GWAS of Deelen et al. (2019) included cases at or beyond the age corresponding to the 90th or 99th survival percentile (11,262 and 3484 cases, respectively), and 25,483 controls whose age at death or at last contact was at or below the age corresponding to the 60th survival percentile. This study was successful in defining new genetic candidates associating with survival extremes, *GPR78* and *CRHR1*.

### Genome/Exome Sequencing of Long-Lived Individuals and Discovery of ‘Compensatory’ Alleles

One limitation of the GWAS approach is that it is only capable of detecting the effect of common genetic variants on aging. Rare variants can be detected through whole-genome or exome sequencing; several studies have used this approach to investigate the genome of long-lived individuals. Not surprisingly, most of the sequencing studies of long-lived individuals that have been performed so far were based on a very small number of samples. Freudenberg-Hua et al. (2014) screened 44 Ashkenazi Jewish centenarians’ genomes and identified some (likely) pathogenic coding variants. In addition, they reported on *APOE* ε4 allele homozygosity in this group. Similarly, Gierman et al. (2014) reported of an individual that despite having a pathogenic variant lived to an age 110 suggesting again a protective mechanism. More recently, Breitbach et al. (2019) sequenced the coding regions of 20 “aging” genes in a cohort of 200 individuals. Based on their physical abilities, these individuals were grouped into “early aging” and “late aging” subsets. The authors did not find the cohorts to be enriched for aging-associated variants. However, extended recent analysis of centenarian and supercentenarian cohorts by Garagnani et al. (2021) detected significant association of rare genetic variations within several loci, in part through comparison to geographically matched replication cohorts and control populations. Their results suggest commonalities among extreme long-lived members of this cohort as sharing variation at gene loci associating with cancer prevention and DNA repair mechanisms as well as broad regulators of pathways including immune functions and Insulin growth factor 1. Combined, the results from these approaches suggest that neither healthy aging nor extremely long life associate with a decreased rate of rare pathogenic variants, potentially indicating the presence of disease-resistance factors in the centenarians (Erikson et al., 2016). These seemingly paradoxical findings – reviewed also by Brooks-Wilson (2013) – fit into a ‘buffering’ paradigm proposed by Bergman et al. (2007). The extremely long-lived people do not lack risk alleles for common diseases.

## Translation of Longevity Model Organisms and Core Aging Pathways

Genetic studies on lifespan have proven to be challenging. While longevity is a defining trait for a given species, the lifespan of individuals is of limited heritability, making analyses more difficult. Exceptional human life span, although a rare phenotype, is likely multifactorial; refined analyses are required to obtain statistically robust genomic signatures of longevity (Zhang et al., 2020) and these have proven elusive. Unlike laboratory models, the effect of environmental variance cannot be controlled in human studies, potentially masking purely biological aging mechanisms. Even laboratory models cannot replicate the complex “environment” of humans; it includes psychosocial, economic, and cultural factors, rather than strictly biological. These human-specific confounders are difficult or impossible to target in traditional model organisms. Despite these limitations, experimentally tractable model organisms have proven invaluable in deciphering the purely genetic contribution to lifespan, including genes and pathways conserved across the tree of life.

The genetic powerhouses of *Caenorhabditis elegans* and *Drosophila melanogaster* have been extensively screened to identify genes with influence on lifespan. The *daf-2* mutant in *C. elegans*, nearly doubling its lifespan (Dorman et al., 1995), was the inaugural discovery into the network of insulin-like signaling, growth regulation, and the protective pathways they modulate. This web entangles the pathways facilitating the beneficial effects of both caloric restriction (López-Otín et al., 2016) and mTOR inhibition (Kennedy and Lamming, 2016). Importantly, these pathways seem universal across metazoans, with consistent results integrating both caloric restriction and mTOR inhibition across classic experimental models, from worms (Lakowski and Hekimi, 1998; Robida-Stubbs et al., 2012) and flies (Partridge et al., 2005; Schinaman et al., 2019), to mice (Zhang et al., 2014; Mitchell et al., 2016), as well as primates (Pifferi and Aujard, 2019). Modulating core regulators in these conserved pathways has been extensively investigated in mice, including genetically heterogenous ones, and generally yields 10–30% lifespan extension (Miller et al., 2011, 2014). This extension, and the concomitant healthspan benefits, would be of undeniable medical value to modern society if they were mirrored in humans. However, this reality has not been born out in genetic analyses in extreme long-lived individuals to date, despite the apparent universality of these pathways. Changes in these pathways, while potent, are likely rare in humans and have not been detectable through GWAS.

## Longevity Extremes Across the Tree of Life

**FIGURE 2:**
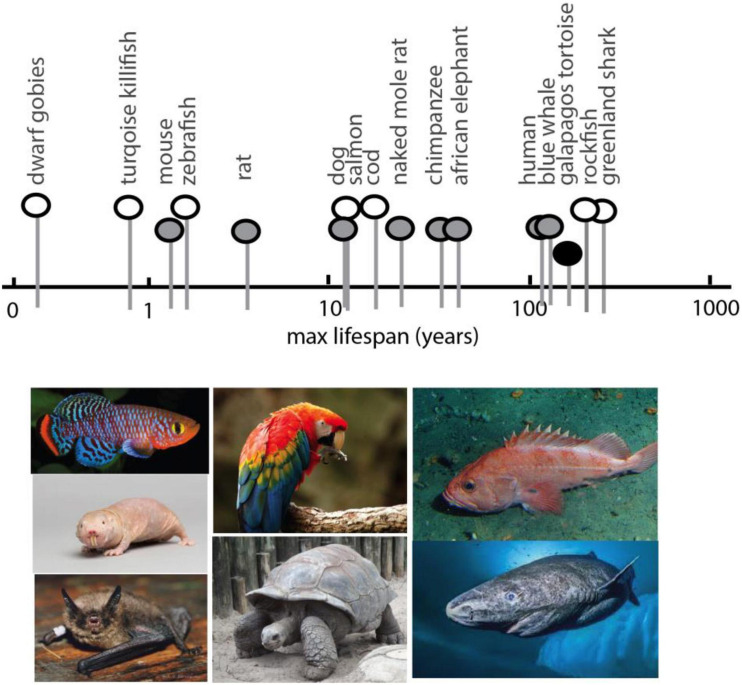
Spectrum of maximum lifespan in vertebrate species: comparative models. Maximum lifespan of select vertebrates showing broad range (over two magnitudes) and diversity. Pictured below are examples of select species exhibiting extreme bounds of lifespan, short to long, for which genomic analysis has been generated. In order, left to right, Turquoise Killifish, Naked Mole Rat (image: National Geographic Creative/Alamy Stock Photo), Brandt’s bat, Scarlet Macaw, tortoise, rockfish, Greenland shark (image: National Geographic). Max lifespan obtained from AnAge (genomics.senescence.info/species/).

Analyses in experimental model systems suggest the existence of a conserved, core set of genes and processes regulating age-related disorders and affecting lifespan (Ma and Gladyshev, 2017; Yanai et al., 2017). However, the changes seen in many of these ‘aging’ or lifespan extending genes are modest compared to the variation between vertebrates (Figure 2). From dwarf gobies with a maximum lifespan less than 2 months (Depczynski and Bellwood, 2005), to the Greenland shark that survives nearly 400 years (Nielsen et al., 2016) (Figure 2), vertebrate genetics possess a remarkable plasticity for life history strategies (Mangel et al., 2007; Ma and Gladyshev, 2017). Despite this variation, constraints in developmental and physiological regulation across vertebrates suggest even disparate lineages are more similar than dissimilar – they employ the same physiological, cellular, and genomic “toolkits” in much the same way. Just as the life- and healthspan effects of mTOR and caloric restriction are widely conserved, phenotypic hallmarks of aging also appear to be shared across disparate lineages (López-Otín et al., 2013) as are the genes involved (Bayersdorf and Schumacher, 2019). The plasticity in longevity may be grounded in the same universal pathways (Mangel et al., 2007). As longevity is an inherent trait of all living things, and one that is inextricably linked to selection for life-history strategies (Luckinbill and Clare, 1985; Zwaan et al., 1995), longevity is also likely a part of core pathways and subject to general constraint across vertebrates.

Because of these selective constraints on the aging and lifespan processes across the tree of life, the diversity of longevities between organisms can be leveraged to decipher the core machinery facilitating it – potentially independent from the mechanisms involved in expression of aging-like pathologies. There is already a rich field investigating unconventional organisms with exceptionally long and short lifespans (Mikuła-Pietrasik et al., 2021), but much of this research is done in relative taxonomic isolation – that is within one or few related lineages. Careful and extensive characterization of these unconventional lineages necessitates this limited sampling, but there will be genetic signatures arising from these analyses that are particular to the group, unrelated to their longevity. These intruding signatures will remain grouped in such an analysis. For instance, the naked mole rat is an extraordinary rodent lineage that can survive past 30 years, fivefold greater than predicted by their size and phylogeny (Buffenstein and Jarvis, 2002). While much has been characterized about their notable aging resistance and the hallmarks thereof (Edrey et al., 2011), it is not feasible to parse specific mechanisms of longevity apart from those qualities of “mole rat” that differ from a comparator such as the mouse. Adaptations to their subterranean lifestyle, diet, colony behavior, and the smorgasbord of general speciation changes will obfuscate any genetic signals facilitating their exceptional longevity. Until recently, the best way to clarify such signals was to lean upon the wealth of longevity knowledge gleaned from conventional genetic models. This focus upon established pathways derived from mutation analyses pigeonholes the results of the association to the large effect changes seen within the artificial contexts and limited genetic diversity of laboratory models. Such limitations on experimental strategies impose stringent blinders; they enable detection of some longevity associated changes, but they remove context which then leads to incongruencies between models. For instance, we know that long-lived Myotis bats maintain their telomeres and have changes in their genetic machinery to do so (Foley et al., 2018), yet telomerase deficient mice take 4–6 generations before their telomeres deteriorate to yield a phenotype (Lee et al., 1998), indicating telomeres are not the dispositive factor of their aging or longevity. The impressive cancer resistance of naked mole rats may be due to modified p16^INK4A^ improving early contact inhibition (Seluanov et al., 2009), but the locus encoding this protein has a unique structure not seen elsewhere and produces a novel isoform (Tian et al., 2015). This is an intriguing adaptation, but it does not extend to the evolution of other long-lived species. These kinds of conundrums demonstrate that a broader system regulating longevity is at play, while the specifics for any single lineage may not necessarily apply elsewhere.

## Comparative Genomics of Longevity

To better reveal the genetic architecture of longevity, we need to tap into the broad diversity of lifespans shaped in evolution. The research into how longevity is modulated has, in a sense, already been performed countless times by Nature. Every lineage with shifts in lifespan is an independent evolutionary experiment in how to modulate longevity through genetic changes. The results of these “experiments” are hereditary, written in the genome, and define the longevity characteristic of a given lineage. The mechanisms facilitating these natural shifts in longevity can thus be read with high-throughput genomic sequencing. Provided sufficient independent occurrences, shared signatures between species can yield statistically significant results, implicating loci with the trait of interest. This strategy for phenotype to genotype mapping was coined “forward genomics” (Hiller et al., 2012; Currie, 2013) and for traits other than longevity, a trove of genomic data has been generated across a wide diversity of taxa. This comparative genomic approach has proven to be a powerful and efficient tool to refine genetic signals of complex phenotypic traits, ranging from echolocation (Parker et al., 2013) to vitamin synthesis (Hiller et al., 2012). As an additional approach, transcriptomes of diverse species have been generated in order to understand changes in gene expression in organisms with varied lifespans (e.g., Seim et al., 2014; Toren et al., 2020; Kulaga et al., 2021). While providing in depth analysis about differential regulation of gene expression, the results of these methods are centered on particular tissues, ages, and genetic contexts, which will introduce confounding variables into multispecies comparisons. For the purposes of this review, we will focus on use of whole genome sequence as a common comparator for change associated with longevity, as it is agnostic to these other variables.

Given the recent expansion of genomic datasets, this experimental approach using sequenced genomes can tap into diverse signals and intersect datasets for powerful meta-analyses. Evolutionary genomic signals include pseudogenization and gene loss, gene family expansion, accelerated evolutionary rate, selection measures, shared amino acid changes, expression levels, and epigenetic marks. These genomic signatures can implicate specific genes, regulatory elements, or entire pathways with the evolution of traits in an unbiased fashion. These approaches have confirmed general genetic constraints between mammals, including humans (Lindblad-Toh et al., 2011) and inspired convergent analyses on a suite of traits. Until recently, these strategies had not been applied to longevity, which has instead centered on isolated pairwise comparisons, limiting their ability to refine specific signals from background speciation changes.

To date, only a handful of comparative genomic studies have been performed with broad sampling for longevity. Convergent evolutionary rate shifts with longevity across 61 mammalian genomes has revealed a set of DNA repair mechanisms and NFκB signaling pathways that are under constrained sequence evolution (Kowalczyk et al., 2020). A similar strategy on the same dataset specifically identified Nucleotide Excision Repair and Chromatoid Body Regulation gene set (Treaster et al., 2021), the latter of which is likely involved in a more general transposon suppression to maintain genomic integrity (Sturm et al., 2015). The constrained sequence evolution these studies reveal suggests those genetic elements are likely critical for the expression of the trait (Roscito et al., 2018). In parrots and other long-lived birds, 344 genes were identified with shared selective pressure and were enriched for cell cycle regulation, RNA splicing, and DNA repair gene sets. The greatest selective pressure was identified on *CAPN10*, also involved in insulin signaling (Wirthlin et al., 2018). In primate longevity, an analysis of parallel amino-acid substitutions found enrichment in wound healing and cardiovascular disorders, while sphingosine 1-phosphate and PI3K pathways were enriched for shifts in evolutionary rates (Muntané et al., 2018). In the broadest study, a Metazoan-wide analysis of 216 eukaryotic genomes revealed the relative size of chaperone networks to generally correlate with longevity (Draceni and Pechmann, 2019), reinforcing a proteostasis theory of longevity. Combined analysis between data types can also enrich true signals from genomic noise. Comparative genomics and transcriptomics in rodents detected 250 positively selected genes with longevity, which when intersected with age related transcriptome changes in naked mole rats revealed enrichments in mTOR, IGF and oxidative stress pathways (Sahm et al., 2018).

However, generally across and within these studies exists a lack of a standard statistical cutoff for significant results and further discussion. While this is not overly problematic in isolation, the idiosyncrasies in determining a “hit” make it difficult to consolidate results for meta-analysis and intersection with other studies. This is further confounded by the intense focus on established aging pathways from experimental models, biasing the discussion toward results that have already been validated. This adds minimal value to the field at large. Novel genes and pathways without established longevity influence are largely ignored, despite often having the most significant scores in the analysis. This strategy is backwards; genomic resources are sufficiently robust that significant results should be revealed within the study itself, and those results should inform validation studies in traditional models. Ignoring these windows of discovery will result in paralysis in efforts to understand how longevity is regulated and how it varies among organisms.

## Epigenomics of Longevity and the Correlation With Lifespan

Maintenance of gene expression over time has been associated with changes in aging. Recent work has tied alteration in gene expression regulation over time to distinct regulation of epigenetic alterations at loci that change as organisms age (Sen et al., 2016). Recently, DNA methylation at cytosine-phosphate-guanosine (CpG) sites has been identified as a specific correlative factor of aging, integrating broader epigenetic regulation with the regulation of promoter sites. These findings led to the definition of sets of genes whose change in expression is tied to methylation level over time and serves as a predictive measure of biological age within a species (Horvath, 2013, 2015). Methylation is a dynamic process during the life of an organism, such that the sequence of genomic DNA does not provide direct evidence of methylation patterns and the consequential lifespan variation. However, the density of CpG sites at promoters within genomes of organisms has been found to be higher in long-lived species (McLain and Faulk, 2018; Mayne et al., 2019). As CpG density is hard written into genomes, regardless of age of the individual, these measures can be read across groups to find loci exhibiting specific variation within long lived species (McLain and Faulk, 2018).

Although changes in DNA methyltransferases can lead to changes in lifespan in experimental models (Sen et al., 2016), it has yet to be experimentally shown that increase in CpG density is causative for changes in gene function tied to increases in longevity. However, the predictive value of this genomic signature provides an interesting genomic signal, one that could be intersected with other signatures to help detail the genetic changes that underlie long lived species.

## All in the Family: The Power of Clade-Wide Analysis to Evaluate Longevity

The quality, quantity, and availability of genomic data is empowering comparative genomic approaches and the generation of new tools to mine variation between and within lineages. However, in many lineages there is a need to increase sampling of related taxa, as often only one representative species may be detailed. As with genetics of humans with extreme lifespan, finding lineages that share particular longevity characteristics increases the power in detection of associated loci. In this case, finding sister species that harbor similar attributes, or have explicitly lost them, allows refinement of potential genetic signals of selection. Additionally, the ability to generate data on the fixation of genetic variation within a species provides an important filter to refine genetic signals, as opposed to focusing on a single individual. The propensity for increased or limited lifespan is a species-level trait and thus, should be shared among individuals of the species. Thus, increased fixation of alleles within the species relative to outgroups having a lack of the lifespan character will aid in detecting *bona fide* genetic signals regulating this trait. We have previously termed such an approach as Phylomapping (Daane et al., 2016), which tracks a variant phenotype as a derived trait within a group. When broader sequencing efforts are combined with the prodigious efforts of naturalists in characterizing organismal diversity, these genomic approaches and analyses can wash away the unique species-specific changes to yield refined signals specific for longevity.

### Leveraging Evolutionary Genesets as Means to Hone Association Studies for Human Longevity and Lifespan

**FIGURE 3:**
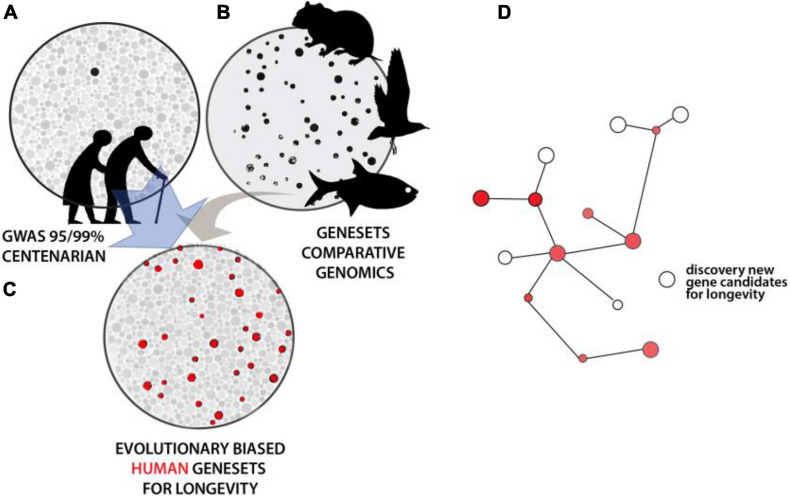
Intersectional analysis to identify conserved genetics of lifespan regulation. **(A)** Illustrative depiction of statistical resolution of genetic association analysis across older human populations and/or long-lived individuals. Strength of shading and size represents level of statistical strength on pathway or gene. Black represents those groups significant at above FDR corrected values. **(B)** Convergence analysis within and across lineages defining broad Gene Sets. **(C)** Using comparative gene sets to focus statistical analysis of human variation reveals broader statistical association within this smaller set (red). These processes or genes underlying these traits are conserved. **(D)** Analysis of hits within known pathways or genetic interactive maps permits flushing out other potential regulators not directly altered in particular lineages.

Results from mutational analysis across eukaryote model organisms have shown unexpected conservation of genes and processes regulating aging. While unique properties exist within particular organisms that modulate these foundational networks, the conservation provides a tool to refine human genetic studies. As noted, GWAS for human longevity metrics suffer from large sample size requirements to obtain statistical resolution due to multiple hypothesis testing across the genome. Assuming that evolutionary genesets for longevity could be generated with confidence, an intersection of them with human variation data would increase the sensitivity of association studies. This would serve as a selective filter to refine the number of loci investigated for association in human populations. Similarly, such evolutionary filters could refine analysis of rare, unique variation within genome sequence data from extremely long-lived cohorts. A similar approach to refine human longevity GWAS used an intersection with age-related disease datasets. This ‘disease-informed’ GWAS helped refine candidates (iGWAS, Fortney et al., 2015), though, it should be noted that this particular strategy would further blur the distinction between aging and longevity as discussed above. The definition of gene sets from evolutionary experiments in longevity, across clades, would similarly empower detection of networks previously hidden under GWAS in human population analyses (Figure 3). Critically, this would be done without bias to the “aging” process *per se* and should instead uncover the genomic cues setting the overall trajectory of our lifespan, and presumably healthspan. As noted, the mechanisms regulating longevity may be broad, with species-specific means of regulation. These extended, shared, mechanisms can be detected through network analyses of the resulting gene candidates, refining the extended biological regulation of these traits (Figure 3D).

## Summary

We have reached a critical point in the generation, access, and analysis of genomic data. Sequencing and characterizing a single genome used to be a massive endeavor. With modern tools, now 267 novel avian genomes or 100 cichlid fish genomes are released in a single paper (Feng et al., 2020; McGee et al., 2020). Additional sampling with a focus on long-lived species will be worthwhile to enrich phylogenomic data that in turn can refine the map between this critical phenotype and its facilitating genotype. New approaches and software to associate this wealth of information with the diversity of longevities present in the natural world continue to expand. These resources can distill genetic regulation of lifespan and how it varies. The system underlying aging is conserved and thus we posit that the genetic regulation of lifespan will also capitalize on common genetic mechanisms. We anticipate that a combination of comparative approaches, of parallel meta-analysis of human longevity genomic signatures along with refined analysis of other exceptionally long-lived vertebrates, will aid in resolving key regulators of enhanced lifespan that have proven to be elusive when analyzed in isolation. Only through such comparative mechanisms will the genetic architecture of longevity be revealed.

## Author Contributions

All authors listed have made a substantial, direct and intellectual contribution to the work, and approved it for publication.

## Conflict of Interest

The authors declare that the research was conducted in the absence of any commercial or financial relationships that could be construed as a potential conflict of interest.

## Footnotes

1 http://genomics.senescence.info/
